# Credit assignment between body and object probed by an object transportation task

**DOI:** 10.1038/s41598-017-13889-w

**Published:** 2017-10-17

**Authors:** Gaiqing Kong, Zhihao Zhou, Qining Wang, Konrad Kording, Kunlin Wei

**Affiliations:** 1 School of Psychological and Cognitive Sciences, Peking University, Beijing, China; 2 Beijing Key Laboratory of Behavior and Mental Health, Beijing, China; 3 Key Laboratory of Machine Perception, Ministry of Education, Beijing, China; 4 Department of Neuroscience & Department of Bioengineering, University of Pennsylvania, Pennsylvania, PA USA; 5 Peking-Tsinghua Center for Life Sciences, Beijing, 100080 China

## Abstract

It has been proposed that learning from movement errors involves a credit assignment problem: did I misestimate properties of the object or those of my body? For example, an overestimate of arm strength and an underestimate of the weight of a coffee cup can both lead to coffee spills. Though previous studies have found signs of simultaneous learning of the object and of the body during object manipulation, there is little behavioral evidence about their quantitative relation. Here we employed a novel weight-transportation task, in which participants lift the first cup filled with liquid while assessing their learning from errors. Specifically, we examined their transfer of learning when switching to a contralateral hand, the second identical cup, or switching both hands and cups. By comparing these transfer behaviors, we found that 25% of the learning was attributed to the object (simply because of the use of the same cup) and 58% of the learning was attributed to the body (simply because of the use of the same hand). The nervous system thus seems to partition the learning of object manipulation between the object and the body.

## Introduction

Humans excel at using tools and manipulating objects. Our dexterous manipulation of objects owes to efficient construction of an internal representation of object property through trial-by-trial learning^1–3^. For example, if movement error occurs during interacting with an object, such as spilling coffee from a cup due to insufficient lifting force, we can rapidly update our estimate of the object weight and change our actions to overcome the error accordingly. It has been proposed that learning from movement errors involves a credit assignment problem: did I misestimate properties of the object or those of my body^4–8^? When interacting with the coffee cup, a misestimate of the properties of our body, e.g. strength of our arm, just as well as a misestimate of the properties of the object, e.g. the weight of a coffee cup, can lead to motor errors. How the nervous system simultaneously updates its estimates of the body and the object during object manipulation is still poorly understood.

Based on the idea of partitioned learning between the body and the object, our recent work has proposed a statistical model to study the credit assignment problem in motor learning^4,5^. The model suggests that only the errors attributed to internal causes (body) but not those attributed to external causes (object) will be used to update the motor system^5,6,9^. Model simulations have provided qualitative explanations for diverse phenomena in motor learning, including generalization, interference, savings and spontaneous rebound^4,9^. Although there is modeling evidence for partitioned motor learning between the body and the object, so far we lack direct evidence about the way the brain solves these kinds of problems.

Previous experimental work has discovered various phenomena suggesting the separation of motor learning when the learner interacts with an object. For example, in a typical motor adaptation paradigm, people learn to reach in an altered force environment with a hand-held robotic handle. The learning can be transferred to free reaching with the handle dismounted; it can also be transferred to reaching with the mounted handle but without applied force. However, the transfer is significantly larger in the latter case even though these two conditions are mechanically equivalent, suggesting that the object itself (aka, the robot handle) “carries” some learning^5,10^. However, all these evidence is indirect.

This lack of direct evidence is largely caused by incompatibility of experimental paradigms. On the one hand, object manipulation paradigms examine the *whether* learned manipulation (e.g., hand grasp or finger manipulation) can be transferred to different scenarios, especially to different objects^11–15^. However, these studies seldom examine *how much* learning is transferred and how it relates to object learning. On the other hand, studies with reaching perturbation paradigms (albeit with a hand-held object) examine how motor learning transfers between limbs^16,17^ or how learning aftereffect is affected when people disengage the object^5,10^. In these studies, between-limb transfer and the reduction in aftereffect by disengaging the object have been hypothesized to be caused by the learning attributed to the object. Again, however, this part of learning has not been quantified or examined in comparison to body learning. Hence, though both object manipulation paradigm and reaching paradigm have found that learning is related to the specific object the learner interacts with, none of the studies quantifies the partitioning of learning between the body and the object within a single experimental framework.

Here we used a novel weight-transportation task to study the transfer of learning between hands as well as between objects. We were able to simultaneously estimate the amount of learning specifically associated with the object (i.e., object learning) and learning specifically associated with the effector (i.e., body learning) with a unified experimental paradigm. In all conditions participants first learned to transport a cup with their right hand. We have three working hypotheses. (1) If the transfer is measured with a second identical cup and with the contralateral hand, the amount of transfer is minimal. This is because that both object learning, associated with the original object, and body learning, associated with the original hand, are absent during the transfer test. The small transfer of learning observed in this condition might merely reflect the learning of the task itself. (2) If the transfer is measured with the second cup but by using the same hand, the amount of transfer should be increased as compared to (1). The increase is a quantification of body learning since this condition only differs from (1) by the use of the same effector. (3) If the transfer is measured by using the contralateral hand but with the same cup, the amount of transfer should also be increased over (1). The increase is a quantification of object learning since the only difference between this condition and (1) is whether the same cup is transported. We found supporting evidence for all these hypotheses. Interestingly, the sum of these three learning components amounts to approximately 100%. Our findings thus provide direct evidence that motor learning, when involving an object, can be roughly partitioned into two independent components that are respectively related to the body and the object, on top of learning of the task itself.

## Materials and Methods

### Participants

We recruited thirty college students (14 females, 16 males, age range: 18–26 years), consisting of 11 participants for *Experiment 1 and* 19 participants for *Experiment 2*. All participants signed an informed consent form and were paid for their participation. Each participant received 100 Renminbi (about 15 dollars) for their participation. They were naïve to the purpose of the experiments. All participants were right-handed, had normal or corrected-to-normal vision without a known history of psychiatric or neurological disorders. The study was approved by the ethics committee of Peking University, and was carried out in accordance with the approved guidelines. We obtained written consent forms from all participants before formal data collection.

### Apparatus and Basic Movement

**Figure 1:**
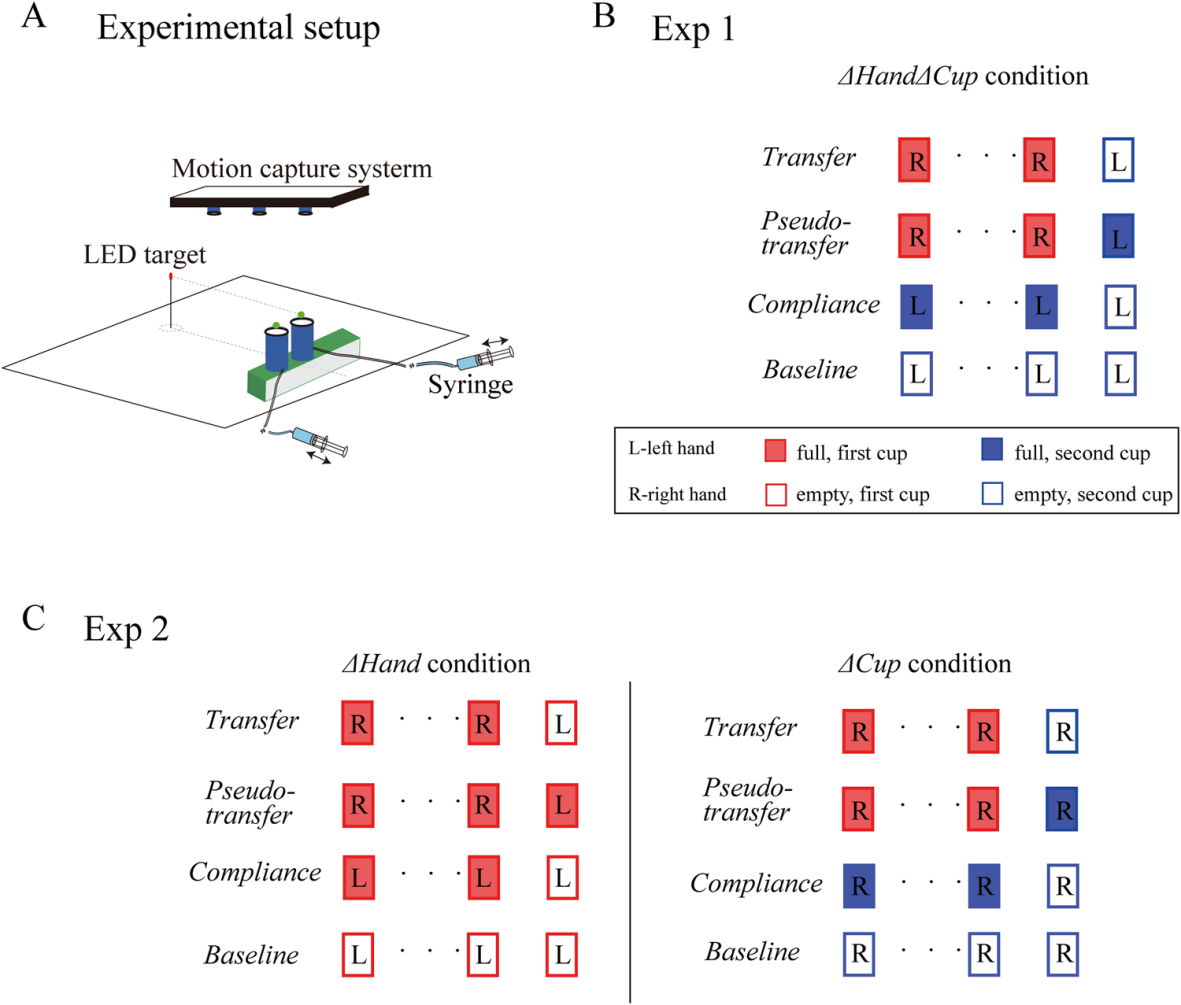
Experimental setup and experimental design. (**A**) Experimental setup. The participant transported a cup, containing varying amount of water, to a LED target after the LED light was lit. The cup was initially placed on a platform and became unsupported once moved. A remote-controlled syringe system changed the amount of water before the trial when needed. (**B**) Schematic illustration of trial blocks used in *Experiment 1*. The four types of trial blocks (*transfer*, *pseudo-transfer*, *baseline* and *compliance*) were randomized in order. The transfer of learning was assessed when both hands and cups were switched (*ΔHandΔCup* condition, only condition in *Experiment 1*). (**C**) Schematic illustration of trial blocks used in *Experiment* 2. *Experiment* 2 utilized similar trial blocks as *Experiment 1* with critical modifications: the transfer of learning was assessed when only hands were switched (*ΔHand* condition), or when only cups were switched (*ΔCup* condition). Squares denote trials within a trial block; color denotes the identity of cups; filled or unfilled squares denote full or emptied cups, respectively. Detailed explanations were listed in Protocols.

The experimental setup was similar to the one that was used in our previous investigations^18^. The seated participant used one hand to transport a cylinder-shaped cup (2.5 cm in diameter and 14.0 cm in height) towards a LED target 15.5 cm straight ahead (Fig. 1A). The LED target and the cup were aligned with the center line of a desk. The cup weighed 220 g when contained a full load of 120 g water. Before each movement, the cup was placed on a platform that was 55.5 cm long, 5.0 cm wide and 4.0 cm high. The platform had a glass top, and its width was equal to the diameter of the cup. Thus, the cup was unsupported once moving. A plastic pin (1.0 cm long) was attached to the cup lid at the same height of the LED target (17.7 cm above the desktop). Participants were required to transport the cup and to make the pin to “touch” the LED target as accurately as possible. A trial started when the target was illuminated, and a beep sound was played by a computer speaker. The participants moved and paused at the target briefly until the LED light was turned off. Then they returned the cup to the starting position and waited for the next trial. Based on their performance, a monetary reward was displayed on a projection wall 1.5 m in the front of the desk.

The typical movement was a straight reach towards the LED target and lasted 900–1100 ms. To prevent slow movements, we played a sharp beep sound if the movement lasted more than 1100ms and canceled the monetary reward. The motion of the cup was measured by an infra-red marker installed at its center top which was tracked by an overhead motion capture system (Codamotion, Charnwood Dynamics, UK). Before each trial, the cup weight could be changed by pumping in and out of the water via a plastic tube running from the cup bottom to a syringe. Participants used a transparent cup. Thus, they could observe the process of water changing and know the weight changes before each movement. The tube was light-weight and flexible as such it did not limit the transportation movement. The action of the syringe was enabled by a linear motor. Both the motor and the LED light were controlled via a programmable circuit board (Arduino Duemilanove) that was connected to a data acquisition PC which ran a customized Matlab program (Matlab 2009b, Natica, MA).

### Protocols

The protocols for *Experiment* 1 and 2 were similar. Each participant performed the weight transportation task in a sequence of trials which were arranged in blocks (Fig. 1B and C).

### Experiment 1

**Figure 2:**
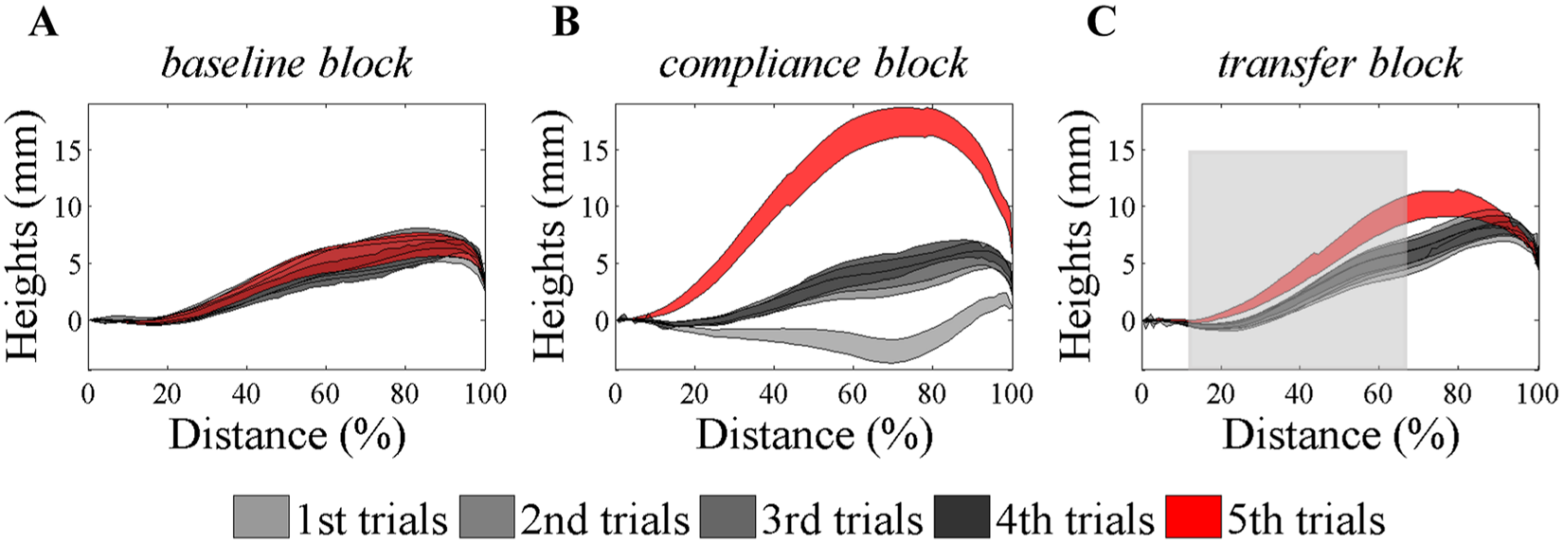
Data from a typical participant of *Experiment 1*. The average trajectory height is plotted as a function of movement distance. (**A**) In a *baseline* block, the left hand moves relatively straight after transporting the emptied second cup for five successive trials. (**B**) In the *compliance* block the left hand is elevated substantially when the cup is unexpectedly emptied after four full-cup trials. The trajectory appears lower in the first trial due to the aftereffect of transporting the mostly likely emptied second cup before the *compliance* block. (**C**) In the *transfer* block, the right hand moves relatively flat in early trials with the full first cup. The left hand transports the emptied second cup in the last trial, resulting in elevated trajectories. It indicates a partial transfer as the elevated trajectory height is still lower than the last trial in the *compliance* block. The gray shade denotes the range covering the duration between the trial-beginning time and the peak-velocity time, and this part of data is used to calculate the trajectory height.

If the learning obtained during object manipulation mainly consists of body learning and object learning as assumed, we shall find minimum transfer of learning when both hand and cup are changed. To test this hypothesis, we conducted *Experiment 1* where participants learned to transport the first cup by using their right hand and then switched to a second identical cup by using their left hand (*ΔHandΔCup*). To quantify people’s learning of transporting weights, we designed four types of trial blocks (Fig. 1B). Each block contained 4, 5, or 6 trials. In a *transfer* block, participants learned the cup weight by transporting the full first cup with the right hand for a random number of consecutive trials (varying between 3 to 5). Then, at the last trial in the block, participants switched to their left hand and transported the second identical-appearance cup. This second cup was initially placed side-by-side with the first training cup on the platform (Fig. 1A). It was moved to the same initial position as the first cup by the experimenter at the beginning of the transfer trial. Our critical manipulation was that the water in the cup might be emptied in the last trial in a trial block, resulting in an elevated hand trajectory. This kinematic change reflected the (mis)estimation of the object weight which had been learned in previous trials (Fig. 2).

Since the last trial in a *transfer* block always invoked a weight change thus its effect could be anticipated by the participant. To overcome this problem, we also designed the second type of trial block called *pseudo-transfer* block. It was nearly identical to a *transfer* block except that its last trial was with the full second cup instead of the emptied second cup. When both *pseudo-transfer* and *transfer* blocks were interleaved, participants could not anticipate a weight change with a transfer action.

The third type of trial bock, *compliance* block, was to measure how compliant the arm was for the object-transportation movement investigated here. During a *compliance* block, the participant transported the full second cup with the left or for a few trials (the number varied between 3 and 5), and then in the last trial, the water was removed from the second cup. Even though participants were aware of water removal, they nevertheless overestimated the weight and moved the cup higher than in previous trials. Thus, using *compliance* blocks, we measured how much elevation would occur if people misestimated the object weight by 120 g. As estimated from our pilot data as well as data from our previous study^18^, the size of height change is a linear function of weight misestimation. We thus were able to obtain the slope of this function as a quantification of the arm compliance.

The last type of trial block was so-called *baseline* block during which we measured the baseline performance without any weight changes, hands changes or cups changes. During a *baseline* block, we asked participants to use the left hand to move the emptied second cup for a block of trials (again with three different block sizes). Typically, the hand trajectories were relatively straight. We only used the last two trials in the *baseline* block to establish the baseline performance.

With the measures of compliance and baseline height, we can map the height changes in the transfer trials into the amount of learning that was transferred after changing hands and objects. It should be noted that a transfer trial was biomechanically identical to its associated baseline and compliance trials. Taking the *ΔHandΔCup* condition as an example, the transfer trial was performed by the left hand with the emptied second cup. Correspondingly, the last trials in a *baseline* block and the *compliance* block also involved the left hand transporting the emptied second cup. Thus, our experimental design enabled us to transform the change in trajectory height in transfer trials into weight estimate errors that were solely due to the transfer of learning.

The experiment included 30 *baseline* blocks, 30 *compliance* blocks, 36 *transfer* blocks, and 18 *pseudo-transfer* blocks. Before formal data collection, participants practiced for 20 trials where cup weight, either full or empty, was randomly assigned. All blocks were randomly interleaved during the experiment and this, together with varying block size, helped the randomization of trials. The inter-trial interval was set at 4 s by the data collection program and the total experiment time (572 trials) was approximately 2 hours. Participants had two mandatory, 5-min breaks upon finishing 286 trials; they could also request rest breaks during the experiment as needed to avoid fatigue.

### Experiment 2

*Experiment 1* established the baseline of the transfer when both object learning and body learning was minimized. *Experiment 2* went a step further to investigate how much learning can be attributed to the body and the object, respectively. To achieve this, we used similar experimental design as in *Experiment 1* with a critical change in the *transfer* blocks: each participant changed either hand or cup during transfer, instead of changing both hands and cups. After learning with the right hand and the first cup, participants switched to the left hand to move the same first cup in the *ΔHand* condition; they kept using the right hand but moved the second identical cup in the *ΔCup* condition. These two conditions were performed in two separate sessions which were collected 24 hours apart.

As either hand or cup was switched, performance baseline and arm compliance were measured differently than in *Experiment 1* (Fig. 1C). For the *ΔCup* condition, the *baseline* block and the *compliance* block only measured the right hand and the second cup since the left hand was not used for the transfer. Instead, only the left hand was measured with the only one cup for the *ΔHand* condition.

In each condition, there were 30 practice trials, 30 *baseline* blocks, 30 *compliance* blocks, 36 *transfer* blocks, and 18 *pseudo-transfer* blocks. Similar to *Experiment 1*, all blocks were randomly interleaved, and block size varied. With a total of 582 trials, each condition lasted for approximately two hours.

### Data Analysis

The participants learned to move a water-filled cup to a target location straight ahead. As they learned about the weight of the cup, their trajectory became straight. An over-estimation of weight will lead to vertical deviations of the trajectory (Fig. 2). We selected the initial part of the trajectory to calculate average height for each trial. This part started from the beginning of the movement, when movement speed exceeded 2.5 cm/s, to the time of peak speed. For analyzing ballistic movements such as reaching, the initial part of the movement up to the time of peak velocity is typically associated with forward control^19^. Taking reaching adaptation in the force field as an example, lateral deviation of the trajectory at the time of peak velocity is regarded as an indicator of learning that is free of the influence of feedback correction. Thus, our choice of using this initial trajectory segment to capture learning is consistent with conventions in motor control studies on reaching. The second half of the trajectory clearly involved feedback correction that did not relate to feedforward estimation of weight and was thus excluded from further analysis. About 1.2% of trials were excluded from analysis due to measurement failure caused by a depleted battery of the motion-capture marker.

We computed the compliance of each arm by taking the difference in trajectory height (*ΔHeight*) between the second last trial and the last trial of *compliance* blocks. These two trials involved an abrupt weight changes of 120 g. For our weight-transportation task, the height changes caused by abrupt weight changes are approximately linear for the weight range tested^18^. We estimated the compliance by dividing *ΔHeight* by the weight change (120 g). Thus, compliance indicates how much height difference is caused by a unit of weight change.

For quantifying the transfer of learning, we could estimate participants’ feedforward estimation of the cup weight. Take the *ΔHand* condition as an example. After practicing with the right hand to transport the first full cup, the participant predicted the cup to be a full one before she picked up the same emptied cup with the left hand. If this is the case, the height change at the transfer trial would be as high as shown in *compliance* blocks which involved the same 120 g weight reduction with the left hand (e.g., 2 cm). Thus, this 2 cm indicates a complete across-hand transfer of previous right-hand learning associated with a full cup. If the participant estimates it to be 40% of a full cup, the resulting height elevation would be 0.8 cm, indicating a 40% of transfer. Thus, trajectory height change at the transfer trial is a direct indication of transfer of learning. By factoring in the individual differences in terms of baseline performance and arm compliance, we can quantify the weight estimate by (*H*_*transfer*_ − *H*_*baseline*_)/*Compliance*, where *H*_*transfer*_ is the height of the transfer trial, *H*_*baseline*_ is the height of the last trial of the *baseline* block, and *Compliance* is the arm compliance measured by *compliance* blocks. This weight estimate was then divided by the actual weight that was learned (120 g) to produce a learning percentage. The resulting percentage indicated how much learning was transferred from the right hand to the left hand (*ΔHand* condition), from the first cup to the second cup (*ΔCup* condition), or from a hand-and-cup combination to a second hand-and-cup combination (*ΔHandΔCup* condition).

Independent t-tests were conducted to compare the learning between experiments. Within-subject comparisons between two conditions were conducted by paired t-tests. The significance level α was set at 0.05.

### Data availability statement

All available data has been presented in the manuscript.

## Results

Participants effectively learned the weight of a hand-held cup by repetitively transporting it. Take a typical participant’s performance in *Experiment 1* as an example (Fig. 2). In the *baseline* blocks, the movement trajectory was almost flat with four consecutive trials with the empty second cup. In the *compliance* block, the trajectory was lowered in the first trial since the preceding trial most likely involved transporting the second emptied cup with the left hand (rare exceptions were those *compliance* blocks that followed *pseudo-transfer* blocks which involved transporting the full second cup with the left hand). Nevertheless, the trajectory became similarly flat when the full cup was transported repetitively for the next three trials. In the last trial of the *compliance* block, the trajectory was elevated substantially when the cup was emptied (the 5^th^ trial in this case). In a *transfer* block, the right-hand trajectory was similarly straight after four trial repetitions; however, the trajectory was elevated slightly in the last trial when participants switched to the left hand to transport the emptied second cup. Note that this height increase was larger when compared with that of the last trial in the *baseline* block though it was much smaller than that of the last trial in the *compliance* block. These last trials in three types of blocks were mechanically equivalent since they all required to use left hand to transport the empty second cup. Their varying heights were listed in Table 1.

**Table 1:** The trajectory heights for the last trials in different trial blocks and estimated *Compliance*.

	Exp 1 *ΔHandΔCup* Condition	Exp 2 *ΔHand* Condition	Exp 2 *ΔCup* Condition
*Baseline* Block Heights (mm)	0.4 ± 0.3	3.3 ± 1.1	3.4 ± 0.9
*Compliance* Block Heights (mm)	5.1 ± 0.7	9.8 ± 1.7	9.0 ± 1.4
*Transfer* Block Heights (mm)	1.2 ± 0.3	6.3 ± 1.6	8.1 ± 1.3
Compliance (mm/kg)	39.3 ± 5.4	58.2 ± 7.0	54.5 ± 5.6

**Figure 3:**
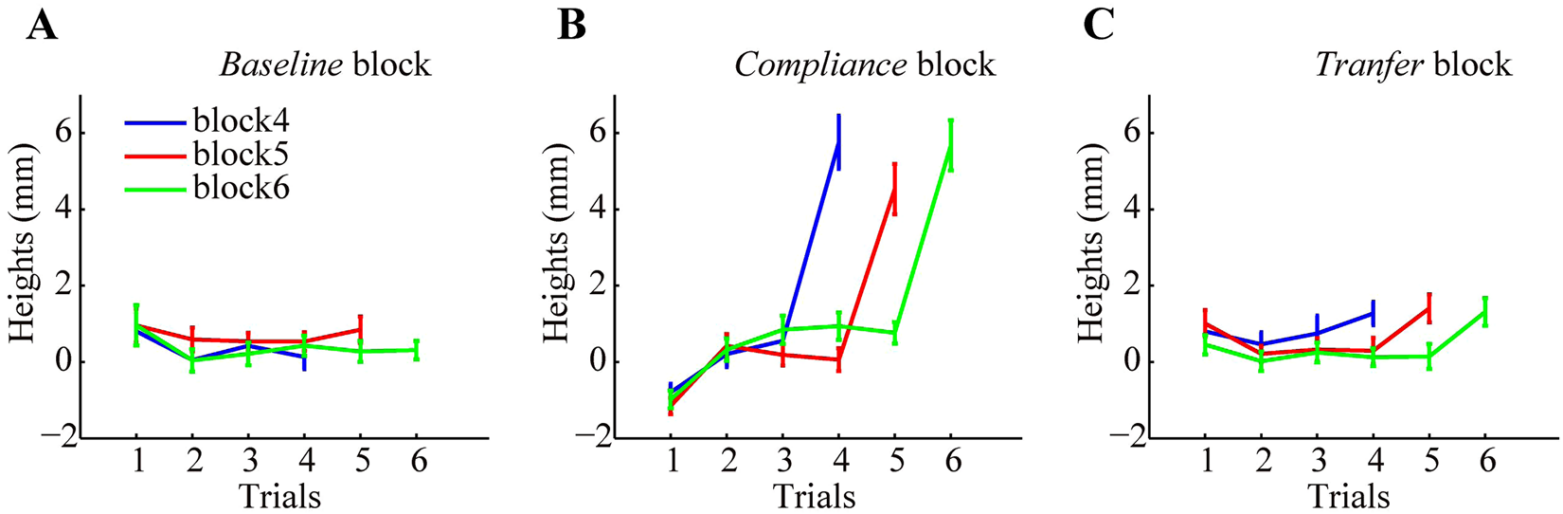
Data from all participants of *Experiment 1*. The trajectory heights are plotted as a function of trial within a block; data from three block types are shown in separate panels (**A**–**C**). Lines of different colors indicate trial blocks of different sizes. A relatively stable height is established with 1 or 2 trials after a weight change. The height increases in the last trial when the second cup weight is emptied, but the increase differs between the *compliance* block and the *transfer* block.

The systematic height changes across trials remained consistent across varying block sizes (Fig. 3). Take *Experiment* 1 as an example again. The last trials in the *transfer* and *compliance* blocks produced increased trajectory heights. The remaining trials in the blocks had similar trajectory heights except that the first trials showed slightly lower heights. This transient effect reflected the aftereffect of occasionally transporting an emptied cup in the immediately preceding trial, which was the last trial in a trial block. Nevertheless, people learned the weight of a hand-held object and quickly converged to a relatively stable height with 1 or 2 trials after a weight change. Thus, our block size was adequately large for learning. These were consistent with previous reports that people can quickly learn inertial properties of the object by hand manipulations^18,20^. In fact, all dependent measures did not show a statistical difference between block sizes; we thus reported average results by collapsing data from different block sizes.

We found that the transfer to the left hand after swapping the cups was a mere 16.3 ± 4.1%. It was significantly above zero (one-sided t-test, *t*_10_ = 3.66, *P* = 0.004, Cohen’s *d* = 1.1) but the effect size was small, considering that participants were fully aware that the second cup was identical to the first during transfer. The baseline height was 0.4 ± 0.3 mm and the compliance of the left arm was estimated to be 39.3 ± 5.4 mm/kg. The average height for the transfer trial was only 1.2 ± 0.3 mm. The minimal transfer thus suggests that most of the learning obtained during this weight-transportation task was not transferable if both the effector and the object are changed.

**Figure 4:**
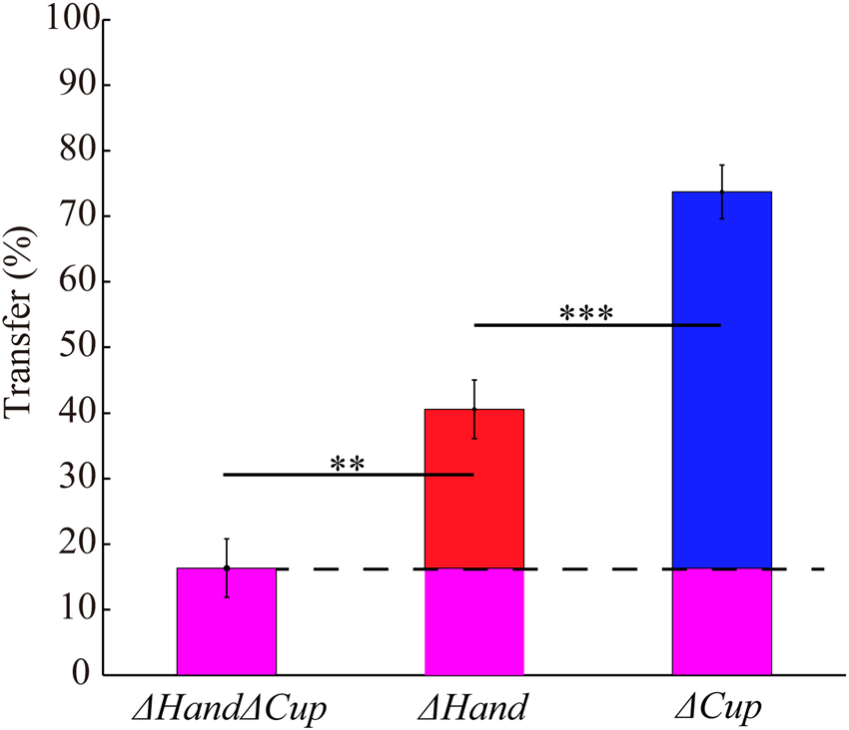
The percentage of transfer of learning in *Experiment 1* and *Experiment 2*. Transferring to a different hand and with a second cup (*ΔHandΔCup*), to a different hand but with the same cup (*ΔHand*), and to a second cup but with the same hand (*ΔCup*) are dramatically different. By removing the baseline level of learning estimated from *ΔHandΔCup*, we can estimate the learning specifically attributed to the cup (*ΔHand*) and the hand (*ΔCup*), as respectively shown in red and blue color bars. ** for *P* < 0.005, * for *P* < 0.05.

In *Experiment 2*, the transfer increased substantially when only one factor, hand or cup, was changed during the transfer tests (Fig. 4). As in *Experiment 1*, we measured the baseline height and the arm compliance to establish participants’ behavioral baseline. The baseline height was 3.3 ± 1.1 mm and 3.4 ± 0.9 mm for the left and the right hand, respectively (Table 1). The compliance was 58.2 ± 7.0 mm/kg and 54.5 ± 5.6 mm/kg, respectively. For *ΔHand* condition, the transfer trial yielded a height of 6.3 ± 1.6 mm, equivalent to a transfer of 40.6 ± 4.5%. This transfer was significantly larger than the transfer observed in *Experiment 1* (independent t-test, *t*_28_ = −3.56, *P* = 0.0013). Note the major difference between these two cases was that the left hand started to pick up the same cup. Subtracting the baseline transfer in *Experiment 1*, the remaining transfer was 24.25 ± 4.5% and significantly above zero (*t*_18_ = 5.42, *P* = 0.00003, confidence interval [14.85 33.65]). This part of learning is attributed to the object itself since the only difference between *ΔHand* and *ΔHandΔCup* was that a second identical cup was used in the latter condition.

Besides quantifying the learning attributed to the object, the data in *ΔHand* condition also demonstrated that there was indeed learning specifically associated with an effector. We compared the elevation of height in the last trials between *compliance* and *transfer* blocks in the *ΔHand* condition. In these blocks, the last trial was the same since both types of blocks required participants to transport an empty cup with the left hand. They differed regarding the history of learning trials: the *compliance* blocks involved learning with the left hand while the *transfer* blocks with the right hand. After transporting a full cup, the last trial showed elevated trajectory height for both types of trial blocks. However, we found that the height elevation was larger in *compliance* blocks (7.0 ± 0.8 mm) than in *transfer* blocks (3.7 ± 1.0 mm), indicating that there was extra learning associated with the effector used for initial learning (*t*_18_ = 4.59, *P* = 0.0002).

For *ΔCup* condition, we performed similar analyses to estimate the transfer of learning that was associated with the effector (Table 1). Compared to *ΔHandΔCup* condition in *Experiment 1*, the transfer of learning increased to 73.7 ± 4.1% for when the same right hand picked up the other cup. Subtracting the baseline transfer, the remaining transfer was 57.38 ± 4.1% (independent t-test against 0, *t*_18_ = 14.008, *P* < 0.00001, confidence interval [48.78 65.99]). This part of learning is attributed to the body since the only difference between *ΔCup* and *ΔHandΔCup* was that a different hand was used in *ΔHandΔCup*. In fact, the learning of the body was larger than the learning of object (*t*_18_ = −4.85, *P* = 0.0001, Cohen’s *d* = 1.1). We also directly compared the elevation of height in the last trials between *compliance* and *transfer* blocks within the *ΔCup* condition. Note, for these two types of trial blocks, participants used the right hand to repetitively transport a full cup before transporting an emptied cup in the last trial. The only difference between them is whether a second cup (same appearance, same weight) was used in the last trial (Fig. 1C, *ΔCup*). We found that the elevation of hand was indeed larger in *compliance* blocks (6.5 ± 0.7 mm) than in *transfer* blocks (5.8 ± 0.7 mm), indicating that switching to a new cup demonstrated less learning and the identity of an object was associated with learning (*t*_18_ = 2.83, *P* = 0.01).

It is interesting to note that the sum of three learning components, i.e., the meta-learning related to the task itself in *Experiment 1*, the body learning and the object learning, was approximately 97.97 ± 5.19% and it was statistically indistinguishable from 100% (*t*_18_ = −0.39, *P* = 0.70).

## Discussion

In our study, participants quickly learned to move the “cup” with a straight movement trajectory. Given that the learning was about transporting a weight, the initial trajectory height of this ballistic movement is a direct indicator of participants’ feedforward prediction of object weight. By analyzing the height changes and factoring in appropriate baseline behavior and arm compliance, we could estimate how much of this learning was transferred between hands and between objects. We found that when participants switched hands to transport a second, identical object, their learning was only minimally transferred (16%, *Experiment 1*). Thus, if the transfer action does not share the same effector and the same object as the original action, the motor system exhibits little transfer of learning obtained during object manipulation even when the participant explicitly knows two objects are identical. Interestingly, the transfer was significantly improved if participants only swapped hands or only swapped objects (*Experiment 2*). By subtracting the baseline transfer estimated from *Experiment 1*, we found that learning attributed specifically to the object was about 24% and the learning attributed specifically to the body was about 57%. Importantly, the sum of three learning components was approximately 100%, suggesting that learning of object manipulation can be indeed partitioned into different functional components. These findings suggest that when learning to transport the cup people assign credits to both the object and their body, and attribute errors differentially to these two sources of variability. Misattribution to the body appears counter-intuitive since the task is about estimating the property of an external object. Our experiments thus put strong behavioral evidence to the claim that the brain attributes errors to the body and the environment during motor learning.

Humans excel at learning inertial properties of the hand-held object during manipulative actions. For instance, simply wielding an object without looking can lead to the accurate estimation of its inertial distribution^21^. In our experiments, the participants indeed exhibited fast learning of weight changes as indicated by quick convergence to the stereotypical movement trajectory. This is why it is intriguing that simple learning of object weight is not completely transferred to the contralateral hand or a second identical object. After all, our task is arguably one of the most common object manipulation tasks during daily life. Our previous investigations have also demonstrated that the familiarity of the task itself facilitates transfer^18,22^. In one study, we found specifically that the weight-transportation task exhibited substantially larger generalization across movement directions when compared to other motor learning tasks involving reaching perturbations^18^. Furthermore, the partial transfer is surprising if we consider that our participants were fully aware that the two objects were identical in weight; but they still failed to transfer their learning between objects and between hands. These findings thus suggest that part of learning acquired during object manipulation is effector-dependent and part of it is object-specific.

This limited transfer, though, is consistent with previous investigations that people are not adept to apply explicit knowledge of inertial property in manual manipulation of the object (e.g., ref.^12^). The limited transfer between hands is also consistent with diverse findings that bimanual transfer is incomplete in motor learning (e.g., ref.^23–26^). The unique contribution of the present study is to quantify the learning components that are specifically associated with the object and with the effector within the same experimental paradigm.

The third learning component, identified as the baseline transfer in *Experiment 1*, might have multiple interpretations. It might be the learning of how to skillfully perform the task which requires coordinating multiple joints to rapidly and accurately transport a small weight to a target. It might be the learning of average weight experienced over the course of the experiment. The latter explanation is less likely since learning appears to be complete within a couple of trials for our task. Furthermore, numerous weight-lifting studies have found that weight estimation, as indicated by finger loading force, is mostly correlated with the immediately preceding trial with no signs of meta-learning^2,3,27^. Hence, even though our current data cannot provide a conclusive explanation, we tend to believe that the small baseline transfer, apart from object learning and body learning, results from learning of performing the task.

The partition of learning between the body and the object is consistent with recent advance in object-manipulation studies investigating sensorimotor memory, which was originally referred to as the memory of an object’s physical properties^27^. Researchers have found that memory of previous actions affects object manipulation^13,28,29^. For instance, after forcefully squeezing an unrelated object people was biased to use a large grip force to lift a familiar object^13^. Thus, even though the squeezing action, performed by the same hand, was not functionally related to the lifting task, it still affected subsequent object manipulation. Thus, researchers extended the concept of sensorimotor memory by including an action-based memory to complement an object-based memory^30^. These two separate memory components are conceptually similar to our definitions of body learning and object learning. While previous studies probed how action-based memory interacts with object-based memory, our study estimates their relative contributions with a novel weight-transportation task.

It is important to acknowledge, though, that the relative contributions of three learning components are likely to vary for different object-manipulation tasks. For instance, reaching studies with force field found substantial object learning since aftereffects were small after letting go the robot^5,8,10^. Dismounting the robot handle is similar to swapping cups in our *ΔCup* condition as both cases involve a change of manipulated object. The small aftereffect in the force-field learning task thus suggests that the CNS only attributes a small likelihood of causality to the body^4,31^. In contrast, our experiment found that 57% of total learning was associated with the body. The force-field learning experiments all involve a robot and virtual reality setting which are rarely experienced in daily life, and thus the CNS might attribute the bulk of the perturbations to the robot, which acts as an object. Instead, weight transportation is a daily practice and in our experiment, a cup is a familiar object with little uncertainty about its inertial property. Thus, we observed much more object learning in our experiment as compared to that in force-field studies.

We postulate that awareness of weight changes, by visual cues and repetitive verbal instructions in our experiment, also affects the credit assignment and the partitioning of learning. Previous studies have found that cognitive contribution influences motor generalization in object lifting^16,32^. Visual information of the object aids the transfer of hand manipulation^33^. Given that the central nervous system (CNS) flexibly learns to act based on the relevance of information^34,35^, we believe that credit assignment and its resulting partitioning of learning will change if people are not aware the weight changes of the object (e.g., with opaque cups).

The exact sequence of trials, or the sequence of weight changes, might also quantitatively affects credit assignment^36^. For instance, if we employed a long sequence of trials without weight changes, as opposed to the current random block design, the body learning and the object learning might have different relative magnitudes. Recently, Fercho and Baugh found that the learning of lifting an object with gradually increased weight can be transferred to lifting a novel weight whereas this transfer is absent if the original learning dealt with an object with abrupt weight change^7^. The authors postulated that the gradually-changed object prompted people to attribute the change to the body, and this body learning then manifested itself during the transfer tests with the second object. Thus, the sequence of weight changes indeed impacts the credit assignment and its associated learning. We postulate that object learning lasts longer as it is akin to the concept of object permanence^37–39^, while body learning changes more rapidly. Early studies on object grasping have found that people retain the knowledge of weight distribution for more than 24 hours^2,40^, while the influence of recent actions only lasts for a brief duration^28^. With accumulating evidence that motor learning consists of components of different time scales^41–43^, our findings suggest that during object manipulation body learning and object learning might be associated with a fast and a slow time scale, respectively. The temporal characteristics of different learning components warrant further investigations within the framework of credit assignment.

Our findings provide a new perspective to study transfer of learning object manipulation. Previous studies on bimanual transfer of weight-lifting actions have found that learning transfers across hands^20,44,45^. However, these studies did not quantify how much learning is related to the object and thus can be transferred. Furthermore, a puzzling finding is that within-hand transfer is impaired if an asymmetric object is rotated after learning^12,46,47^. These findings led to propositions that learning to manipulate an object is based on a hand frame of reference^12,48^ or multiple grasp-specific representations^46^. Our credit assignment account gives an alternative but a simpler explanation: only body learning is transferred between objects; if rotated, the asymmetric object has an opposite weight distribution and demands an opposite hand manipulation as previously learned. Thus, the transfer should be negative, i.e., previous body learning should interfere with subsequent manipulation of the object. This is exactly what has been found^12,47,49^.

Our findings have important implications for studies on motor learning and motor rehabilitation. People have proposed that when interacting with the external world, the nervous system has separate modules for the body and different objects^50,51^. Multiple models of different objects offer the brain both flexibility and robustness in performing motor tasks and this account has been supported by recent behavioral and neurophysiological findings^52–54^. Our findings, along with accumulating evidence, extend this theory by suggesting that learning should be decomposed into separate representations/modules for the body and the object. This insight is particularly relevant for motor studies with interactive objects, e.g., hand-manipulated object and robotic manipulandum. Furthermore, the object can also be viewed as a property of the environment whose representation is continuously updated by the CNS^9^. Thus, tasks that involve an altered environment (e.g., visuomotor transformation by virtual reality or prism goggles) should also be considered as governed by separate representations. In the realm of motor rehabilitation, virtual reality and robots are gaining increasing popularity with the premise that learning can be transferred to daily life. It is thus important to elucidate the factors underlying the partition of motor learning since only the body learning can be transferred to other contexts when the patients disengage virtual reality and robots.
